# Rapid and Continuous Cryopreservation of Stem Cells with a 3D Micromixer

**DOI:** 10.3390/mi13091516

**Published:** 2022-09-13

**Authors:** Lin Ding, Sajad Razavi Bazaz, Jesus Shrestha, Hoseyn A. Amiri, Sima Mas-hafi, Balarka Banerjee, Graham Vesey, Morteza Miansari, Majid Ebrahimi Warkiani

**Affiliations:** 1 School of Biomedical Engineering, University of Technology Sydney, Sydney, NSW 2007, Australia; 2 Micro+Nanosystems & Applied Biophysics Laboratory, Department of Mechanical Engineering, Babol Noshirvani University of Technology, P.O. Box 484, Babol 47148-71167, Iran; 3 Cell Science Research Center, Department of Cancer Medicine, Royan Institute for Stem Cell Biology and Technology, Isar 11, Babol 47138-18983, Iran; 4 Regeneus Ltd., Paddington, Sydney, NSW 2021, Australia; 5 Institute of Molecular Medicine, Sechenov University, 119991 Moscow, Russia

**Keywords:** cryopreservation, 3D printing, microfluidics, stem cells, bioprocessing industry

## Abstract

Cryopreservation is the final step of stem cell production before the cryostorage of the product. Conventional methods of adding cryoprotecting agents (CPA) into the cells can be manual or automated with robotic arms. However, challenging issues with these methods at industrial-scale production are the insufficient mixing of cells and CPA, leading to damage of cells, discontinuous feeding, the batch-to-batch difference in products, and, occasionally, cross-contamination. Therefore, the current study proposes an alternative way to overcome the abovementioned challenges; a highly efficient micromixer for low-cost, continuous, labour-free, and automated mixing of stem cells with CPA solutions. Our results show that our micromixer provides a more homogenous mixing of cells and CPA compared to the manual mixing method, while the cell properties, including surface markers, differentiation potential, proliferation, morphology, and therapeutic potential, are well preserved.

## 1. Introduction

Stem cell cryopreservation is the last step of the stem cell production process before storing and delivering the cell products to the clinics for stem cell treatment. This process is carried out by mixing cryoprotectants (or cryoprotecting agents, CPA) and cells in a defined ratio. At the moment, dimethyl sulfoxide (DMSO) is the most commonly used CPA in MSC cryopreservation [1,2], due to the high cell viability and conservation of stem cell properties after thawing. DMSO permeabilises the cell membrane, minimises dehydration of the cells, and prevents overconcentrating of solutes inside the cells [3,4,5,6] to protect cells from water crystallisation. However, exposure to a high concentration of DMSO or long-term exposure to DMSO cause detrimental osmotic shock to the cells and might be lethal to them [7,8,9,10]. Therefore, minimising the exposure of DMSO to cells [11] and controlled mixing of DMSO and cells is critical for protecting cells during the freezing process.

Currently, the standard technique for mixing large volumes of MSCs with CPA is robotic arms technologies, such as the robotic filling line from Aseptic technologies. In this system, DMSO is pre-mixed with cells in a bulk manner before aliquoting the cell–DMSO solutions into the cryopreservation tubes. This means 100% DMSO is added into the cell solution in a large mixing tank, where direct exposure to a high concentration of DMSO is, as described above, lethal to the cells. Then the cells are stored in the large mixing tank for up to 2 h until all the cells are gradually filled into cryopreservation tubes and moved to the temperature-controlled freezer. DMSO changes cell permeability within minutes [12], and the uneven mixing and temporary storage under room temperature cause significant batch-to-batch differences [11] in the MSC products. They can potentially affect the clinical outcome of the cell treatment. The only alternative method, also the most common method used in laboratories, is manual mixing. Manual mixing imposes human error which increases the chance of contamination. It has exceptionally low throughput, which also leads to batch-to-batch quality differences at a large scale, and it does not meet the current Good Manufacturing Practice (cGMP), where no human intervention is allowed. Therefore, there is an enormous need for novel technologies that can introduce CPA into cells homogenously [11].

Microfluidic devices can be the perfect solution to mix CPA and cells. They are miniaturised channels used to manipulate fluids inside the channels, possessing low-cost, disposable, and simple operation features and can be easily implemented in different conditions [12,13]. There were attempts to implement sheath flow-added straight channels to replace the media of the cells to CPA-added media. The advantage of these devices is cell washing happens while CPA is added to the cells, yet the throughputs are only µL/min level [10,14], which has no potential in industrial application. Among the microfluidic devices, micromixers are miniaturised fluid-mixing devices that have attracted significant attention in recent years due to their efficiency and high throughput mixing of a small amount of liquids [15,16]. They are extensively used in biomedical diagnosis, food and pharmaceutical industries, drug development, and chemical synthesis [17,18]. Broadly, micromixers are divided into active and passive categories. Active micromixers rely on external energy input, such as thermal, acoustic, magnetic, or electric fields, to disturb the fluids and achieve high mixing efficiency. Active mixers have a relatively simple structure and high mixing efficiency. However, compared to passive micromixers, active micromixers broadly require stirrers, air interface, fine-tuning, integrated electrodes, heaters, and external energy sources, which have low energy efficiency and could be detrimental to some biological samples [17,19,20]. Therefore, passive micromixers are preferred by the industry. Passive micromixers have complex structures and geometry inside the channels compared to active ones. The complex structures and maximised interfacial area enhance molecular diffusion and chaotic advection, which vividly improve fluid mixing [19]. In general, passive mixers offer unignorable benefits, including better accessibility, low operation cost, high reliability, and easy integration with other systems [17,18,19,20], although 3D passive micromixers are sometimes more complicated to fabricate than active micromixers [21,22,23].

**Figure 1:**
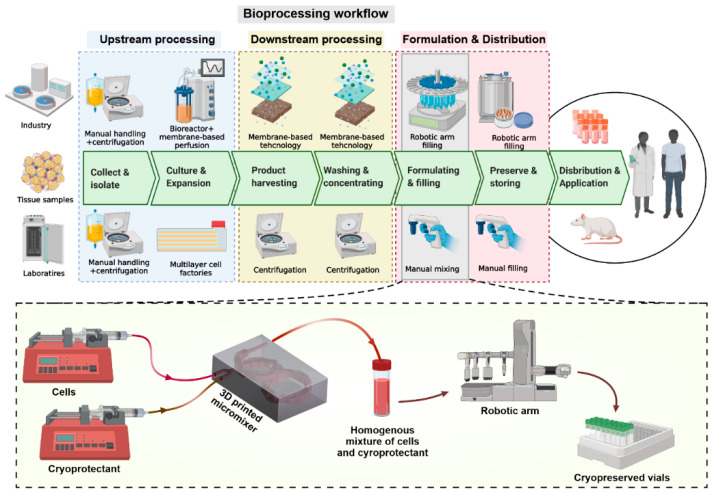
Stem cell production process and the potential application of our proposed micromixer during this process. The micromixer can be either integrated into the current robotic arm system to perform homogenous mixing of stem cells and cryoprotectants or used separately in laboratories to increase the speed of cell–cryoprotectant mixing.

Therefore, to optimise the stem cell cryopreservation process in the industry, here, we present a novel 3D-printed microfluidic mixer for efficiently mixing CPA with MSCs in a high-throughput manner (Figure 1). The micromixer was firstly characterised by mixing food dye and water, and mixing cells with cytoplasmic staining. The properties of cells cryopreserved by mixing with DMSO in the micromixer were then compared with manual mixing controls. The results show that the presented setup preserved the critical stem cell criteria [2], including viability, growth rate, and phenotype of the cell, without changing the properties and differentiation potential. Although we demonstrate the application of the device here with a small sample size (<5 mL cells), the 3D-printed micromixer has the potential to perform large-scale cryopreservation. It can also be easily integrated into other microfluidic systems [6,13] or the current industry system at a low cost.

## 2. Materials and Methods

### 2.1. Micromixer Design and Fabrication

The micromixer was fabricated as previously described [13]. Briefly, SolidWorks 2018 × 64 (SolidWorks Corporation, Waltham, MA, USA) was used to design the micromixer. The micromixer was then converted into an STL file and fabricated with Clear Resin V4 (RS-F2-GPCL-04) via a Form2 SLA 3D printer (Formlabs, Somerville, MA, USA). The layer thickness of the print was set at 50 µm to ensure parts are of high quality. After printing, the micromixers were carefully removed from the build plate, and the support was cut off by a scalpel and scissors. The device was then washed thoroughly with isopropyl alcohol (IPA) three times to prevent the uncured resin from blocking the channel and dried by an air nozzle. Lastly, the device was further cured by a 450 nm UV light in a UV-curing chamber for stabilisation. The channel dimension is shown in Figure S1.

### 2.2. Governing Equations and Boundary Conditions

Laminar flow holds as the Reynolds number ($Re=\frac{\rho Ud}{\mu }$) does not exceed 700 for the present case. Therefore, the incompressible, isothermal, and steady-state Navier–Stokes equation (Equation (1a)), coupled with the continuity equation (Equation (1b)), governs the flow field:

(1a) $$\rho \left(u\cdot \nabla \right)u=-\nabla P+\mu \nabla^{2}u,$$

(1b) ∇·u=0,

where fluid density and dynamic viscosity are assumed to be $\rho =998\;\mathrm{kg}/m^{3}$ and $\mu =8.9\times 10^{-4}\;\mathrm{Pa}\cdot s$, respectively. First, the flow field is solved for the velocity vector field, u, and pressure, P. Then the concentration distribution, c, is calculated by the stationary convective–diffusive transport equation (Equation (2)):

(2) $$\left(u\cdot \nabla \right)c=D\nabla^{2}c,$$

with $D=1\times 10^{-10}\;m^{2}/s$ being the diffusivity coefficient between the fluids. The inlets are assigned the uniform velocity boundary conditions with a 1 to 9 ratio for the CPA ($\Delta c=1\;\mathrm{mol}/m^{3}$) to cell media. The outlet is set to zero pressure. The remaining boundaries are considered to be no-slip and no-flux walls. COMSOL Multiphysics, a commercially available software based on the finite element method, was utilised to simulate and predict the laminar mixing of the solutions within the micromixer. Two modules of laminar flow and transport of diluted species were used to carry out the numerical simulations. The P1 + P1 was used for the discretisation of the fluids, and the quadratic was used for the discretisation of the transported diluted species. The whole domain was solved in two parts: first, the laminar flow module was solved, then, by the extracted values for velocity and pressure, the transport of the diluted species module was solved. The GMRES (Generalised Minimum RESidual) solver was utilised for both laminar flow and the transported diluted species module. An unstructured grid was generated for the entire domain with several boundary layers. Independent grids tests were provided in Figure S2.

### 2.3. Mixing Index

To evaluate the mixing performance of the device, the mixing index or M.I. is defined by assessing the standard deviation of the concentration (σ):

(3) $$M.I.=1-\sigma =1-\sqrt{\frac{1}{n}\sum_{i=1}^{n}\times {\left(\frac{c_{i}-\underline{c}}{\underline{c}}\right)}^{2}},\;$$

where n is the number of sampling points, $c_{i}$ denotes the molar fraction of samples, and $\underline{c}$ represents the totally mixed molar fraction [24].

### 2.4. Micromixer Characterisation

The mixing efficiency was experimentally characterised in two ways. The first characterisation was done by passing red food dye and DI water through the micromixer in a 1:9 mixing ratio at different flow rates, as previously described [25]. Briefly, pictures were taken after the fluids became steady at the inlet and before the outlet of the micromixer. The pictures were then analysed by ImageJ (National Institute of Health, National Institute of Health, Bethesda, MD, USA); values of the grayscale images were recorded and normalised via the following equation:

(4) $$I_{i}=\frac{I_{i}^{*}-I_{min}^{*}}{I_{max}^{*}-I_{min}^{*}}$$

where $I_{i}^{*}$ is the value of the outlet image intensity, and $I_{max}^{*}$ and $I_{min}^{*}$ are the maximum and minimum values of intensity of the fluid at the inlet images. Then, $I_{i}$ was used to calculate mixing efficiency ME:

(5) $$ME=\sqrt{\frac{1}{N}\overset{N}{\underset{i=1}{\sum }}{\left(\frac{I_{i}-\bar{I}}{\bar{I}}\right)}^{2}}$$

and

(6) $$ME_{no\;mixing}=\sqrt{\frac{1}{N}\overset{N}{\underset{i=1}{\sum }}{\left(\frac{I_{min,max}-\bar{I}}{\bar{I}}\right)}^{2}}$$

where N is thenumber of pixels in the images, $\bar{I}$ represents the totally mixed molar fraction, and $I_{min,max}$ are 0 and 1, respectively. These two values were then used to calculate the experimental mixing index:

(7) $$M.I._{experimental}=1-\frac{ME}{ME_{no\;mixing}}$$

and the degree of experimental mixing efficiency in these channels was compared with numerical results obtained using Comsol Multiphysics.

The second characterisation was done by mixing cells with a membrane-staining dye. CellBrite^®^ Cytoplasmic Membrane Dyes (Biotium, Fremont, CA, USA) were firstly characterised by mixing with cells at different concentrations (0.1 µL to 5 µL/million cells/mL). The dye stained the cells seconds after they came into contact. Therefore, a low concentration that cannot stain all the cells (0.5 µL/mL) was used to evaluate mixing efficiency, since even mixing stains more cells than uneven mixing (although uneven mixing might stain some cells with higher fluorescent intensity). The selected concentration was then added to DMSO (Gibco, Australia), and the dye-added DMSO was mixed with cells either manually or through the micromixer at a 1:9 ratio. Then the cells were collected and analysed by microscope and a CytoFLEX LX flow cytometer (Beckman Coulter, Brea, CA, USA) to record the level of staining of individual cells. The results were compared with manually mixed CPA and cells, and statistical differences were compared by two-way ANOVA in Prism (GraphPad, San Diego, CA, USA).

### 2.5. Cell Culture

P7 MSCs were kindly provided by Regeneus. The cells were cultured on T25 (Falcon, Chicago, IL, USA) flasks with α-MEM (Gibco, Australia) supplied with 10% human platelet lysate (In vitro Technologies, Australia) and passaged 2 times before this experiment to eliminate the impact of cryopreservation. Then, the cells were harvested at 90% confluence with a TrypLE enzyme (Invitrogen, Waltham, MA, USA) and centrifuged at 500× *g* for 5 min to replace the enzyme with fresh culture media (90% α-MEM and 10% human platelet lysate) at a concentration of 1 million cells/mL.

### 2.6. Experimental Setup

The experimental setup is illustrated in Figure 1A, and the experiments were perfomed under room temperature. The cells and DMSO solutions were loaded into 3 mL syringes (BD plastic) and connected to the device with Tygon tubings (John Morris, Sydney, Australia). Two syringe pumps (Fusion Touch, Chemyx Inc., Stafford, TX, USA) were used to hold these two syringes separately, and the flow rate ratio was fixed to 9 to 1 for cells and DMSO syringes. The cells were collected from the outlet by a cryopreservation tube and placed in Mr. Frosty (Sigma Aldrich, Sydney, Australia) inside the −80 °C freezer immediately. This allowed the temperature of the sample to decrease in a controlled rate. Five control groups were made by adding and pipetting 100 µL of DMSO in 900 µL cells in fresh culture media. One group was put into Mr. Frosty (Thermo Scientific, Waltham, MA, USA) immediately after mixing, two groups were left at room temperature (RT) for 30 min and 60 min, and the last two groups were left in a 4 °C fridge for 30 min and 60 min before being put into Mr. Frosty. This was to mimic the cell damage caused by long-term incubation with DMSO. All groups of cells were left in the freezer for one week before thawing for cell characterisations.

### 2.7. Cell Characterisations

The cells were defrosted by the standard rapid thawing method, which was to place and stir the tubes in a 37 °C water bath until defrosted [26]. The cryopreserved cells were characterised by their post-thaw viability, growth rate, morphology, stem cell surface marker staining, trilineage differentiation, and immunoproteins staining. The thawed cells were stained with live and dead staining (Abcam, Cambridge, UK) for 10 min in a dark room and proceeded through a flow cytometer to evaluate the viability. To assess the growth rate, the thawed cells were seeded in a 6-well plate at a concentration of 10,000 cells/mL and the media was replaced the next day to wash off dead cells. Images of 5 random positions in each well were taken every day to measure the cell doubling time, and, three days later, the cells were stained with live and dead staining. Fluorescent pictures were taken by an IX 70 fluorescent microscope (Olympus, Tokyo, Japan).

To confirm the stem cell properties via surface marker staining, the thawed cells were harvested by TrypLE after culturing and passaging three times. They were washed by DPBS twice, and fixed with 100 µL methanol (Sigma Aldrich, Sydney, Australia) in a −20 °C freezer for 10 min. After that, the cells were washed twice with DPBS and resuspended in 100 µL DPBS with 10 µL anti-CD73-FITC, anti-90-FITC, and anti-105-PE antibodies (miltenyi biotec, Sydney, Australia) and incubated at a 4 °C fridge for 1 h, according to the manufacturer’s protocol. Then the cells were proceeded through a CytoFLEX LX flow cytometer and the results were analysed in CytExpert (Beckman Coulter, Brea, CA, USA).

Differentiation potential of the cells was confirmed by culturing the cells in 6-well plates with osteogenic, chondrogenic, and adipogenic differentiation media (Gibco, Australia) for 21 days, with the media replaced every three days. On the 21st day of culture, the cells were washed three times with DPBS, before being fixed by 70% ethanol (osteogenic cells) and 4% paraformaldehyde for 1 h at room temperature. Then, the cells were washed three times with deionised water and stained with Alizarin Red (osteogenic cells), Oil Red (adipogenic cells), and Alcian Blue (chondrogenic cells, all staining purchased from Sigma-Aldrich, Sydney, Australia), respectively. The osteogenic and adipogenic cells were stained for 1 h before washing the remaining staining off with deionised water, and the chondrogenic cells were stained overnight. The cells were imaged by a bright-field microscope (Olympus, Tokyo, Japan).

The therapeutic properties of the cells were verified by priming the MSCs by cytokine and staining the change of expression level of the immunoproteins. The MSCs were seeded in 6-well plates after thawing. When the cells reached 50% confluence, priming media containing 10 ng/mL TNF-α protein and 100 ng/mL IFN-γ protein (Stem cell technologies, Cambridge, MA, USA) were added to the wells, and incubated in the incubator for 24 h. Then, the cells were harvested and resuspended in 1 mL cold DPBS and stained with HLA-G and iCAM antibodies (Biolegend, Wangara, Australia) for 1 h. The cells were then proceeded through a CytoFLEX LX flow cytometer, and the results were analysed in CytExpert.

## 3. Results

### 3.1. Design Principles of the Micromixer

Figure 1 illustrates the principle of micromixing and how the flows are guided through the micromixer structures. The design consists of two back-to-back circles with average diameters of 18 mm and manifolds of d=1.5 mm twisted through the channels’ winding shape. The initial stage begins with introducing the stem cell solution and the CPA into their inlet manifolds. The micromixer’s volume and footprint are ~668 mm^3^ and 11 × 22 × 41 mm^3^, respectively.

Mixing at the micro-scale is often achieved by a common technique called split and recombination (SAR) [25,27]. Here, a group of five different SAR components, namely one-sided, middle-sided, and double-sided screws, are arranged sequentially. Figure S1 shows the shape of the SAR units comprised of at least one division and reunion of flow that undergoes a swirling path to induce vortices and mass transport. Moreover, the double-sided unit takes advantage of the third dimension to ensure efficient 3D mixing. After conducting a preliminary analysis, the best combination of the units and their order was selected.

**Figure 2:**
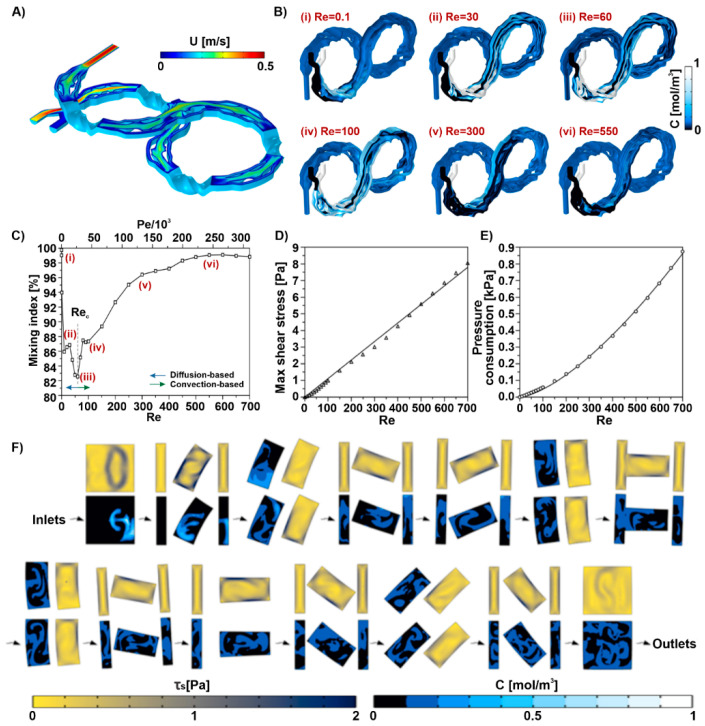
Simulation results demonstrate the mixing performance, pressure, and shear stress in the micromixer. (**A**) Velocity changes on the mid-plane at Re=550. The alteration of pathways and the complex geometry induce velocity shifts and, thus, chaotic and secondary flows. (**B**) The concentration distributions on the micromixer’s walls at different Re, representing the mixing mechanisms. (**C**) The calculated mixing index at various operational Reynolds numbers. The corresponding Re to minimum M.I. is known as the critical Reynolds ($Re_{C}$), representing the mixing mechanism’s transition from diffusion to convection. (**D**) The maximum shear stress during the mixing as a criterion for cell viability. Although it exceeds the physiological limit of 6 Pa, short exposure time, as well as a small coverage area, allow most of the cells to stay alive at the end of the mixing cycle. (**E**) Pressure usage at different Reynolds numbers highlights the relatively small pressure requirement. (**F**) The changes in concentration and shear stress distribution at cross-sections inside all 12 units at Re=550. The dispersion of 0.1 mol/m^3^ entails the effectiveness of the device.

The unique design of the 3D micromixer allows the velocity to change effectively across the fluid’s path required to achieve high-performance mixing. Figure 2A depicts the flow shifts along the channel and the lateral migration of the mass at a high Reynolds number of 550. The joints between each unit show that fluid tends to follow its previous path due to the fluid inertia and the micromixer’s curvature. This is known as the Coanda effect, and in the present case, it significantly enhances the mixing efficiency as a consequence of strong chaotic flow.

### 3.2. Effect of Reynolds Number on Mixing Performance

The mixing efficiency over a wide range of Re is explored to evaluate the performance of the micromixer and the mixing mechanism. As Re increased from 1 to 60, the effect of convection becomes predominant over the diffusion. There is also around 5% fluctuation in the M.I. between Re=30−100, which is due to the nature of the flow where the two inlets meet and create disturbances. The geometry of the junction and the flow rate ratio might reflect on the flow state, creating discontinuities in the mixing performance. Figure 2B illustrates the dispersion of concentrations along the channel walls for these Reynolds numbers. As shown in Figure 2C, the mixing index is above 94 percent for Re<1, regardless of the operational condition. These relatively low-speedflows, which also correspond to low Peclet ($Pe=\frac{Ud}{D}$), are the most suitable condition for the diffusion-based mixings, where lateral transport occurs within the time frame of the main flow. That is, Re=0.1 in Figure 2B shows a gradual gradient of concentration as a manifestation of the diffusion mechanism.

Before the critical Re of 60, mixing is accomplished mainly through diffusion. Most passive micromixers use geometric-based manipulations of fluids, such as curvature-induced Dean flows [2] and out-of-plane translation, which are associated with convection and proportional to $U^{1.63}$. Accordingly, as the Reynolds number exceeds 100, convective mixing outweighs diffusion. Thus, M.I. experiences a sharp growth for Re ranging from 100 to 550, after which it almost remains constant. Here, the convection-based concentration contours in Figure 2B further confirm the proposed micromixer can achieve efficient mixing within a shorter mixing length. Overall, the proposed micromixer can efficiently maintain a mixing quality above 82% in a vast range of Re.

### 3.3. Pressure and Shear Stress inside the Micromixer

Shear stress level within the channel is an important factor that needs to be evaluated, especially when proposing a new system that involves sensitive biological materials, including stem cells. High shear stress may jeopardise the cell viability at high Re. Considering a simplified model, the shear stress in a square duct is given as [28]:

(8) $$\tau_{s}=\mu \cdot \dot{\gamma }=\frac{6\mu Q}{h^{3}}=\frac{6\mu^{2}}{\rho h^{2}}Re,$$

where $\dot{\gamma }$ is the shear rate, Q is the flow rate, and h is the height of the square cross-section. It is evident from Equation (8) that the analytical shear stress linearly depends on the flow condition or Re with a slope of $\alpha =\frac{6\mu^{2}}{\rho h^{2}}=7.62\times 10^{-4}$ as a function of fluid properties and geometry.

In the peripheral blood system, the reported values for $\tau_{s}$ are approximately less than 0.1 Pa considering both small and large capillaries. Meanwhile, in the cardiovascular system, $\tau_{s}$ is on the order of 6 Pa for physiological or exercise conditions [29,30]. This can have a considerable impact on the metabolic response, behaviour, viability, or stem cell fates if they experience the stress for a significantly long time, i.e., longer than an hour [31,32]. The total length of the micromixer is approximately 135 mm, allowing the cells to pass through the micromixer in ~15 s at a 1 mL/min operation flow rate. This is far shorter than the above-mentioned conditions. As a result, the maximum shear stress is calculated over the range of studied Re and the trend is shown in Figure 2D. As discussed earlier, the shear stress was expected to change by Re linearly. However, the value of the α in the numerical model was calculated 14.6-fold higher using the equation provided for the straight duct. This difference stems from the complexity of the geometry under the study that causes high-efficiency mixing but at the cost of slightly higher shear stress.

Input energy is another important factor to consider when using high-throughput microfluidic devices for cell applications. Micromixer design is the crucial factor in controlling input energy [25]. Pressure consumption defines the trade-off between the throughput and efficiency. The amount of pressure consumed in the proposed micromixer is illustrated in Figure 2E. Owing to the small secondary flows at Re<100, a linear relationship was obtained for the pressure usage, whereafter it was estimated via a quadratic function as a result of large vortical patterns. It was then postulated that the proposed mixer design required a relatively low pressure of less than 0.9 kPa to mix the biological solutions efficiently and safely.

The cross-sectional concentration laminae and shear stress can better represent how the tortuous design of the micromixer induces swirls and vortices. Figure 2F illustrates the shear stress and concentration gradient on the cut-planes intersecting each screw-shaped component and perpendicular to the main flow. The chaotic flow created in every region of the micromixer implied that the cells and reagents can be efficiently mixed (the shear stress can be also presented by the vorticity (∇×u)). Flow stretched and folded from the moment the two solutions met each other, and the effect continued along the channel most dominantly within the screw-shaped units. Moreover, the highest stress grew mainly near the walls because of the high-velocity gradient, yet this effect is small in the proposed micromixer due to the large cross-sectional area of the channel.

### 3.4. Mixer Characterisation

The mixing efficiency of the micromixer was first characterised via mixing of food dye and water. To test the device performance, we used three different flow rates while maintaining the flow rate ratio at 1:9 (Figure S3). The results show that the micromixer can fully mix the samples, with higher than 85% mixing efficiency across all flow rates. At very slow flow rates, the dominant fluid regime was Stokes and samples had enough time to properly mix with each other. At high flow rates, the fluid behaviour became chaotic. Given the special geometry of the micromixer, it could still mix the samples well at extremely high flow rates and in a non-laminar flow regime.

**Figure 3:**
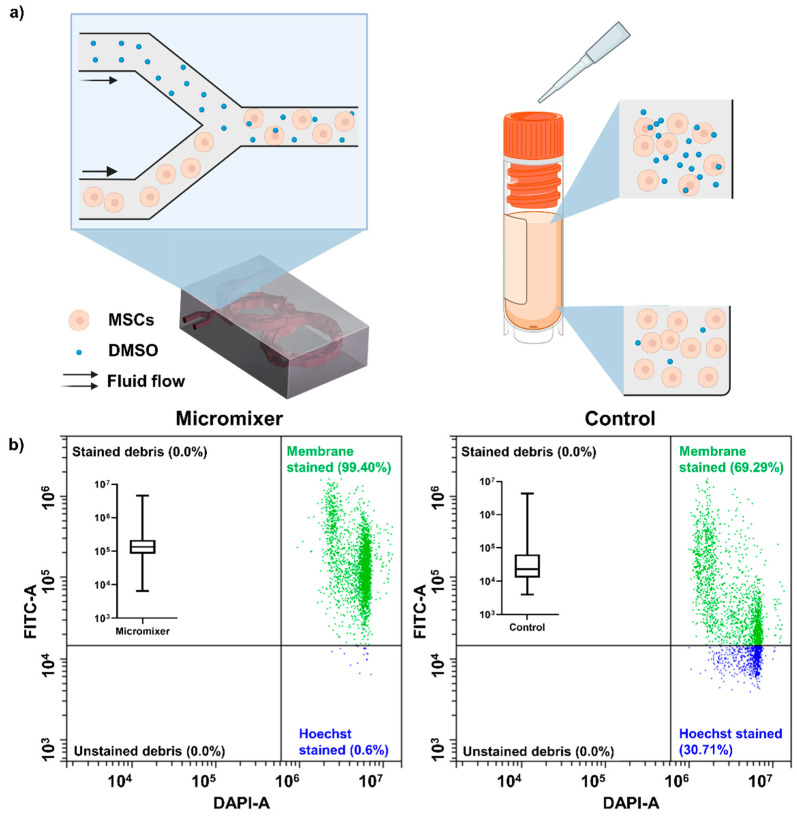
Cytoplasmic dye was added into DMSO to verify the homogeneous mixing of the micromixer experimentally. (**a**) Schematic illustration of the mixing strategies between micromixer and manual mixing. The micromixer introduced DMSO into cells at a constant ratio, while manual mixing faces challenges of uneven distribution of DMSO at the beginning. (**b**) The flow cytometry results of cells mixed with cytoplasmic dye–DMSO in two methods: micromixer (left) and manual mixing (right). The inserted box plots show the level of FITC staining of each cell were summarised in box plot. The results show that the cells in the micromixer groups were more evenly stained than the manual mixing group, and the difference of distribution was significant (*p* < 0.001).

Figure 3 shows mixing performance characterised by mixing cells and cytoplasmic dye. The lipophilic cytoplasmic membrane dye stains the cells immediately after contact (Figure S4). Therefore, reducing the concentration of staining to a level that is not enough to stain all the cells can show the mixing effectiveness of the micromixer. Figure S4 shows the staining of different concentrations of cytoplasmic dye on 1 million cells/mL. Above 1 µL/mL concentration, all cells were stained immediately after adding the dye. When the dye concentration was reduced to 0.5 µL/mL, some cells remained unstained even after 20 min of incubation. Theoretically, a micromixer should provide more homogenous staining of cells due to the high mixing efficiency and the continuous mixing feature. This was proved by mixing dye-added DMSO and cells in the micromixer; the idea is illustrated in Figure 3a. Figure 3b shows the flow cytometry results of membrane dye mixing. The cells processed by the micromixer have a smaller area of distribution, and almost all cells (99 ± 0.13% cells of over 3000 events) were stained with the dye, while the manual mixing control has only 67 ± 4.11% of cells stained. The box plots in Figure 3b show the cells in the micromixer group were stained more homogeneously than the manual mixing control group, since the stained cells distributed in a smaller region than the control group in *x*-axis (FITC-stained). The difference between the two groups was significant (*p* < 0.001). Figure S5 shows the microscopic images of cells from these two groups, and the results align with Figure 3b.

### 3.5. Cell Characterisation

Viability, morphology, proliferation, differentiation potential, and surface protein expression are the key parameters for evaluating the effect of cryopreservation methods for MSCs [2]. Therefore, passage 9 MSCs were used to verify the cell properties after cryopreservation.

**Figure 4:**
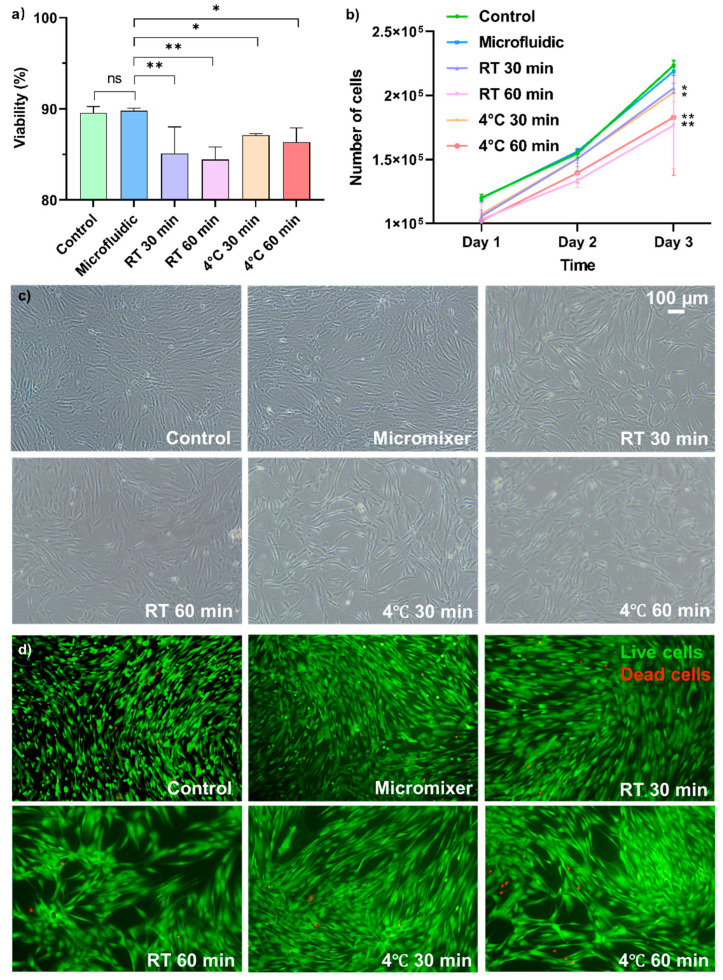
Cell viability and proliferation rate after exposing cells to DMSO under different conditions before cryopreservation. (**a**) Post-thaw viability of cells in different groups. Viability of each group is: 89.79 ± 0.28% for the microfluidic group, 89.55 ± 0.69% for the instant mixing control group, 85.10 ± 2.92% and 84.44 ± 1.38% for the RT 30- and 60-min groups, and 87.09 ± 0.19%, 86.35 ± 1.56% for the 4 °C 30- and 60-min groups. * indicates the significance between groups and n.s. indicates non significant. (**b**) Growth rate of cells after seeding them in 6-well plates for 3 days; the micromixer and instantly preserved control groups had a significantly higher growth rate than the other groups (*p* < 0.001). (**c**) Cell morphology and (**d**) live and dead staining in 6-well plates on day 3 show mixing the cells and CPA in the micromixer does not damage the cell viability, morphology, and phenotype.

Figure 4a,b show that the cells’ viability and proliferation rate were similar to the instant cryopreserved control group, indicating that the micromixer and handling process did not damage the cells. Meanwhile, the RT and 4 °C groups showed time-dependent reduction of cell viability and growth rate, and the microfluidic group had significantly higher viability and proliferation rate compared to them. Figure 4c compares the cell morphology of all groups after three days in culture. The micromixer-processed group showed similar size and spindle, fibroblast-like shapes to the instant cryopreserved control group, indicating the morphology, structure, and integrity of the cells were not altered. Live and dead staining of cells after 3 days in culture show that the attached cells were healthy cells with no long-term damage caused by the device (Figure 4a). There was no change in cell morphology after exposure to DMSO for 30 and 60 min; however, the number of dead cells in the 30- and 60-min incubation groups increased compared to the control group, which was stored in the −80 °C freezer immediately after mixing. Figure 4c,d also show an increased cell size in the RT and 4 °C groups, indicating potential loss of stem cell pluripotency [33,34].

**Figure 5:**
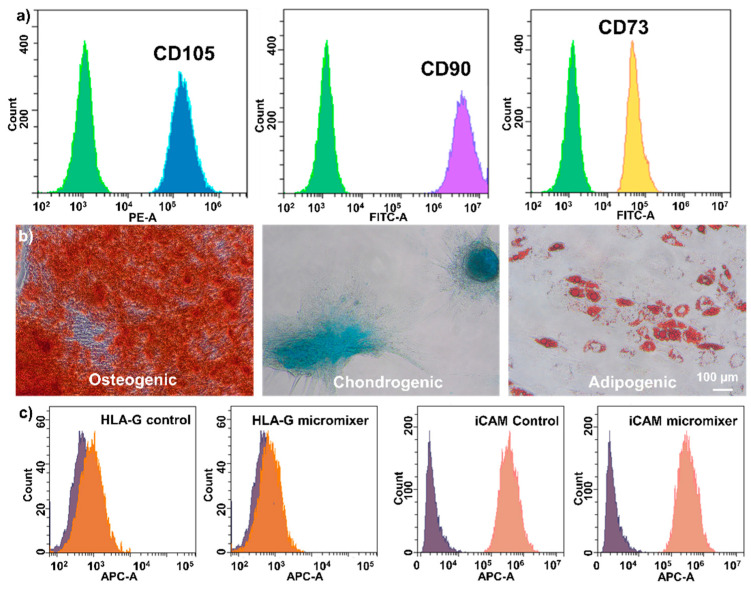
Characterisation of stem cell properties after thawing. (**a**) The micromixer-cryopreserved cells preserved their MSC identities, expressing all three MSC surface markers. (**b**) The osteogenic, chondrogenic, and adipogenic cells showed that the micromixer-cryopreserved cells also maintained the differentiation potentials. (**c**) Comparison of the expression levels of the surface therapeutic markers in the control and microfluidic groups, showing the therapeutic properties of the MSCs passed through the micromixer were not hampered.

The stem cell properties were verified by surface marker staining, trilineage differentiation, and surface immunoproteins staining. After three passages, the cells were harvested and stained by fluorescence-tagged antibodies, and the results show that the MSCs preserved their stem cell properties (Figure 5a). Trilineage differentiations showed the cells preserved their differentiation potentials (Figure 5b), as indicated by the Alizarin Red-stained mineralised matrix of the osteoblasts, Alcian Blue-stained glycosaminoglycan complex of chondrocytes, and Oil Red-stained lipid vacuole of adipocytes. HLA-G and iCAM are two surface-expressed immunoproteins responsible for interacting with the immune system. HLA-G is an immune prohibitor that should remain low after priming, while iCAM is an immunity promotor that increases the immune activity after priming. Figure 5c shows that the micromixer-processed cells express the surface proteins at a similar level to the control group, indicating the therapeutic potential of the MSCs is well-preserved.

## 4. Discussion

The debate on using cryopreserved or fresh-cultured MSCs for patient treatment has continued for many years. CPA were found to damage the cell viability, integrity, structure, and therapeutic potential when they were used at a high dosage or left with the cells for an excessive period [1,2,35,36]. These damages caused by cryopreservation are one of the reasons for unsuccessful clinical trials [37]. For example, low cell viability MSCs failed to treat the chronic inflammatory disorder in a clinical study [38], and cryopreservation-induced cytoskeleton damage reduced the T cell activation capacity of MSCs [39]. Luckily, the cell damage caused by cryopreservation is reversible. There were attempts to reverse or avoid the cell damage caused by cryopreservation by recovering and culturing the stem cells for a few passages before administration or use the MSCs without cryopreservation [2,35,40]. However, these approaches increased the cost due to using expensive clinical-grade, xeno-free cell culture consumables, which limited the therapeutic window (the treatments need to be carried out according to the growth and confluence of the cells) and accessibility to small clinics, and created the need for cell culture-related technicians.

Thus, cryopreservation of MSCs remains one of the standard procedures in MSC treatment, and more optimisation of this process is urgently need [37]. Especially in the bulk mixing process, a high concentration of DMSO added into the cells results in the instant formation of large pores on the cell membrane [41] and a large proportion of cell death [7,8,9], which can be the main reason for the batch-to-batch differences of the products in the industry at the moment. Here, we used a novel micromixer to continuously mix up CPA and MSCs to reduce the batch-to-batch product quality differences of the cryopreservation process. We employed a new method to demonstrate the homogenous and continuous micromixer mixing by processing cells with cytoplasmic dye added to DMSO in the micromixer. Our results show that the cells processed by our micromixer have a significantly more homogeneous mixing than manual mixing group (Figure 3). In this paper, we used a total flow rate of 1 mL/min to showcase the application. This flow rate sits at a diffusion-based dominant regime in the mixing index graph (Figure 2C), where the mixing efficiency is high enough to create a homogenous sample at the channel output. Viability of cells stays as high as 90% after passing through the device and cryopreserved controlled-rate freezing for one week (Figure 4a), and the growth rate (Figure 4b), morphology (Figure 4c), and in-culture viability (Figure 4d) are indistinguishable from the control. Since this is a small lab-scale experiment, we believe that a difference in viability can be observed when it comes to the large scale, where bulk mixing creates higher shear stress and cells are exposed to high concentration DMSO for a longer period, as shown in Figure 4. The well-preserved stem cell identity was verified by the three MSC surface marker staining (Figure 5a). the M’Cs’ differentiation potential was confirmed by trilineage differentiation (Figure 5b) and, most importantly, the immunoproteins expression level in response to stimulations was unaffected (Figure 5c). These results indicate that the device and the whole handling process did not mitigate the cell properties. DMSO was found to exert heat while mixing with cells [35,36], which might cause damage to the cells in large volumes. The small mixing volume and large surface-to-volume ratio of the mixer can potentially help release the heat [42], and although it is hard to prove, the cell property measurement results indicate that there is no damage caused by fusion heat. The manufacturing method is robust and can tolerate high pressures up to 250 psi or more without any trace of channel disruption or crack propagation [43]. The shear stress applied by this micromixer was as small as 1 Pa for Re lower than 100, which is very small compared to the shear stress generated by the stirring tank used in the stem cell industry (which can go up to 300 Pa) [40,44,45]. This shear stress is also smaller than other microfluidic devices which are considered to be harmless to the cells [46]. Yourek, et al. [47] showed that inducing differentiation of MSCs via applying continuous shear stress requires 24 h exposure of cells under shear stress. In this experiment, the cells could pass through the micromixer within ~15 s, which minimised the chance of cells being damaged or altered by the system. In addition, the potential of this device is not limited to mixing DMSO and stem cells; it can be used to mix cells with other reagents at any given ratio.

The micromixer used in this experiment possesses multiple advantages compared to the current CPA mixing methods and other micromixers. Compared to other micromixers, this 3D passive micromixer does not require external energy to operate, reducing the cost of operation. Compared to other passive micromixers, this 3D micromixer has a short total length and big enough channels to reduce the potential damage to the cells. Compared to the current industrial CPA mixing strategies, this micromixer has high mixing efficiency, and it can reach 99% at high Re, which ensures the complete mixing of DMSO with cells in a short time in the correct ratio. The simple setup, designs, and small footprint of the device gives a low fault rate to the system and it can be easily integrated into any current industrial system in a closed-up manner by connecting the devices and bioreactors/cell containers with a peristaltic pump. This closed system meets the cGMP needs, which requires no human interference in the cell-manufacturing process. The device has the potential to increase the throughput to fit industrial applications by increasing the flow rate and paralleling multiple devices. As shown in Figure 2C, the mixing index of the micromixer stays above 90% across multiple flow rates.

## 5. Conclusions

This paper described a novel micromixer to mix stem cells and CPA for the stem cell industry. This device offers a labour-free, rapid, evenly, and continuous mixing, which the current methods cannot achieve. We used cytoplasmic dye to show the micromixer can mix cells and CPA more homogenously than manual mixing, with minimum cell damage introduced during the process. Our results show that cells processed by the micromixer have well-preserved viability, morphology, and cell growth right after thawing. Stem cell properties such as surface markers, differentiation potentials, and immunoproteins expressions that respond to stimulants were well preserved. These results strongly support the fact that our device can be adapted by the bioprocessing industry and has the potential to be used for numerous clinical applications.

## Data Availability

The data presented in this study are available in insert article or supplementary material here.

## Supplementary Materials

The following supporting information can be downloaded at: https://www.mdpi.com/article/10.3390/mi13091516/s1, Figure S1: The design and dimension of the micromixer; Figure S2: The grid independence test; Figure S3: The results of food dye mixing with DI water characterise the mixing efficiency of the micromixer in a real case; Figure S4: Characterisation of cytoplasmic dye concentration before using it for a mixing experiment; Figure S5: The micromixer mixing group and manual mixing group control cells after 15 min of staining.
