# Supplementary material for: Rapid and Continuous Cryopreservation of Stem Cells with a 3D Micromixer
Source: Micromachines (Basel). 2022 Sep 13;13(9):1516. doi: 10.3390/mi13091516 (PMC9500807; doi:10.3390/mi13091516)
Supplement: Supplementary file 1 [file micromachines-13-01516-s001.zip › micromachines-1875729-supplementary.pdf]

Rapid and Continuous Cryopreservation of Stem Cells with a 3D Micromixer

Lin Ding [1], Sajad Razavi Bazaz [1], Jesus Shrestha [1], Hoseyn A. Amiri [2,3], Sima Mas-hafi [2,3], Balarka Banerjee [4], Graham Vesey [4], Morteza Miansari [2,3] and Majid Ebrahimi Warkiani [1,5,*]

1 School of Biomedical Engineering, University of Technology Sydney, Sydney, NSW 2007, Australia
2 Micro+Nanosystems & Applied Biophysics Laboratory, Department of Mechanical Engineering, Babol Noshirvani University of Technology, P.O. Box 484, Babol 47148-71167, Iran
3 Cell Science Research Center, Department of Cancer Medicine, Royan Institute for Stem Cell Biology and Technology, Isar 11, Babol 47138-18983, Iran
4 Regeneus Ltd., Paddington, Sydney, NSW 2021, Australia
5 Institute of Molecular Medicine, Sechenov University, 119991 Moscow, Russia
* Correspondence: majid.warkiani@uts.edu.au

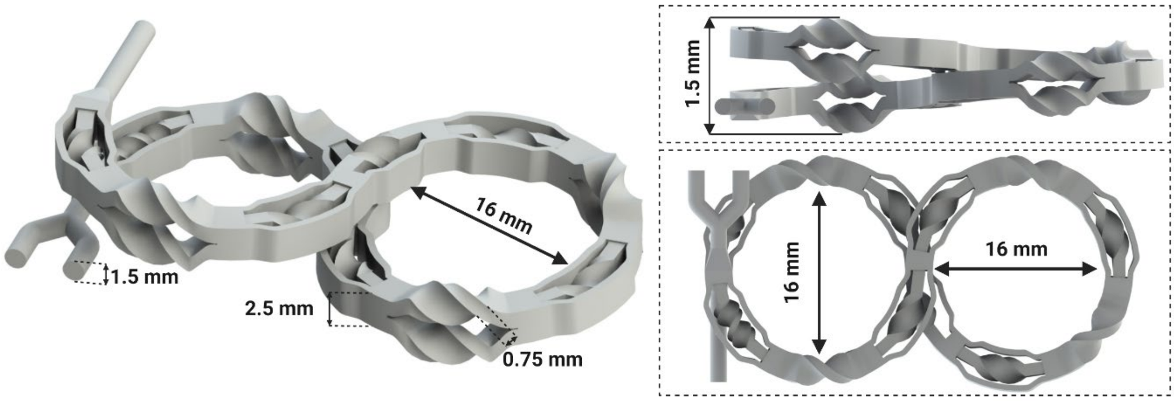

Figure S1. The design and dimension of the micromixer. Mixing units used in the proposed design each has unique geometry and functionality, hence flow characteristic.

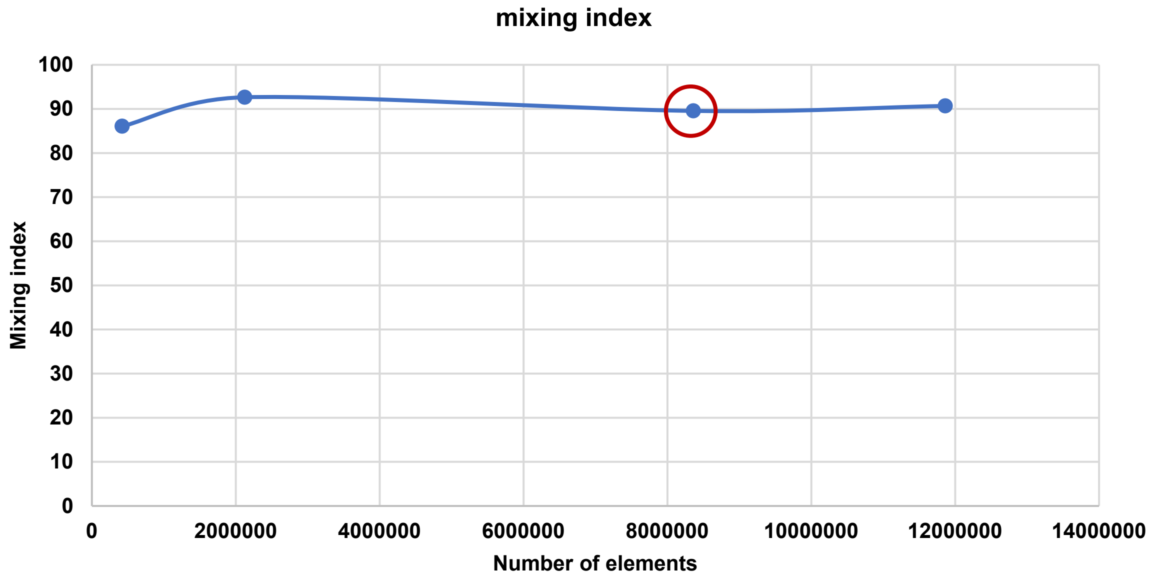

Figure S2 Grid independence test for simulating the micromixers.

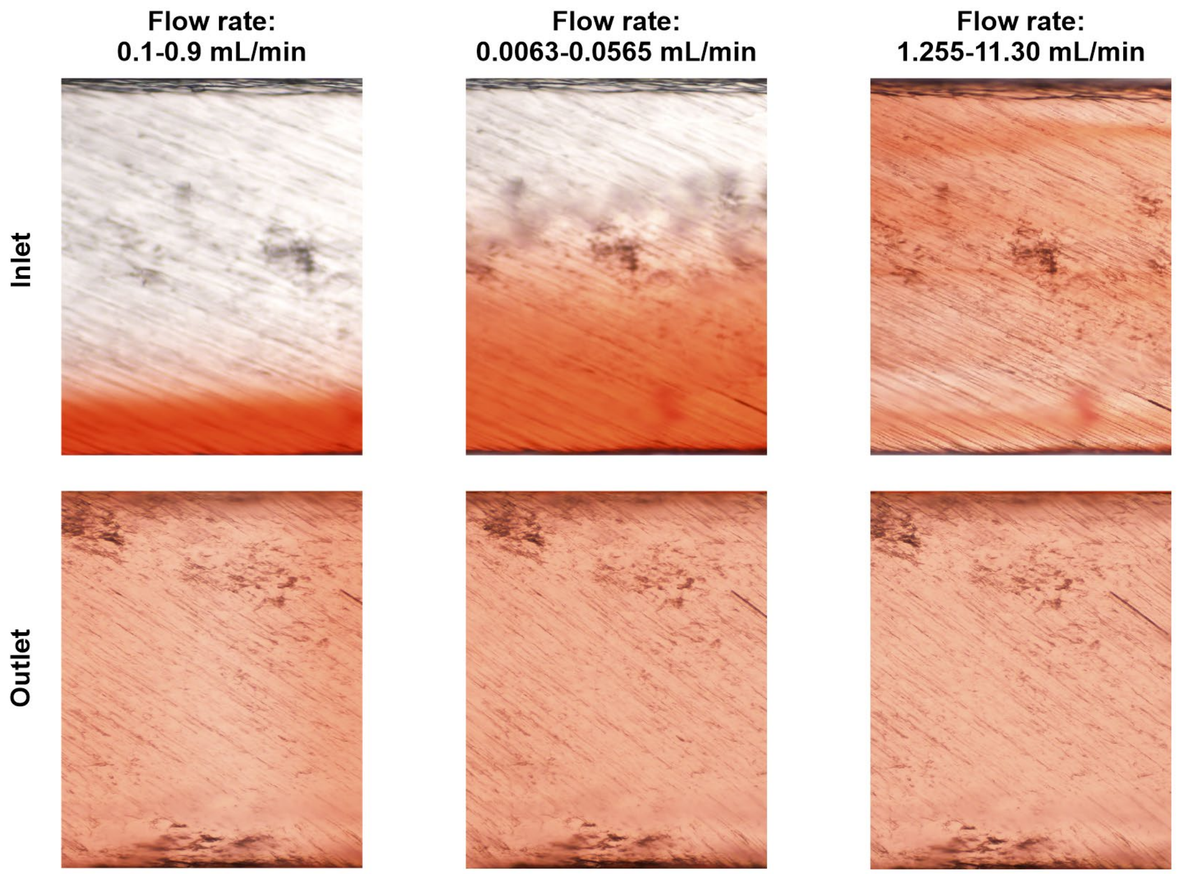

Figure S3 The results of food dye mixing with DI water characterise the mixing efficiency of micromixer in real case. The figures of the outlet showed homogenous mixing of food dye across different flow rates.

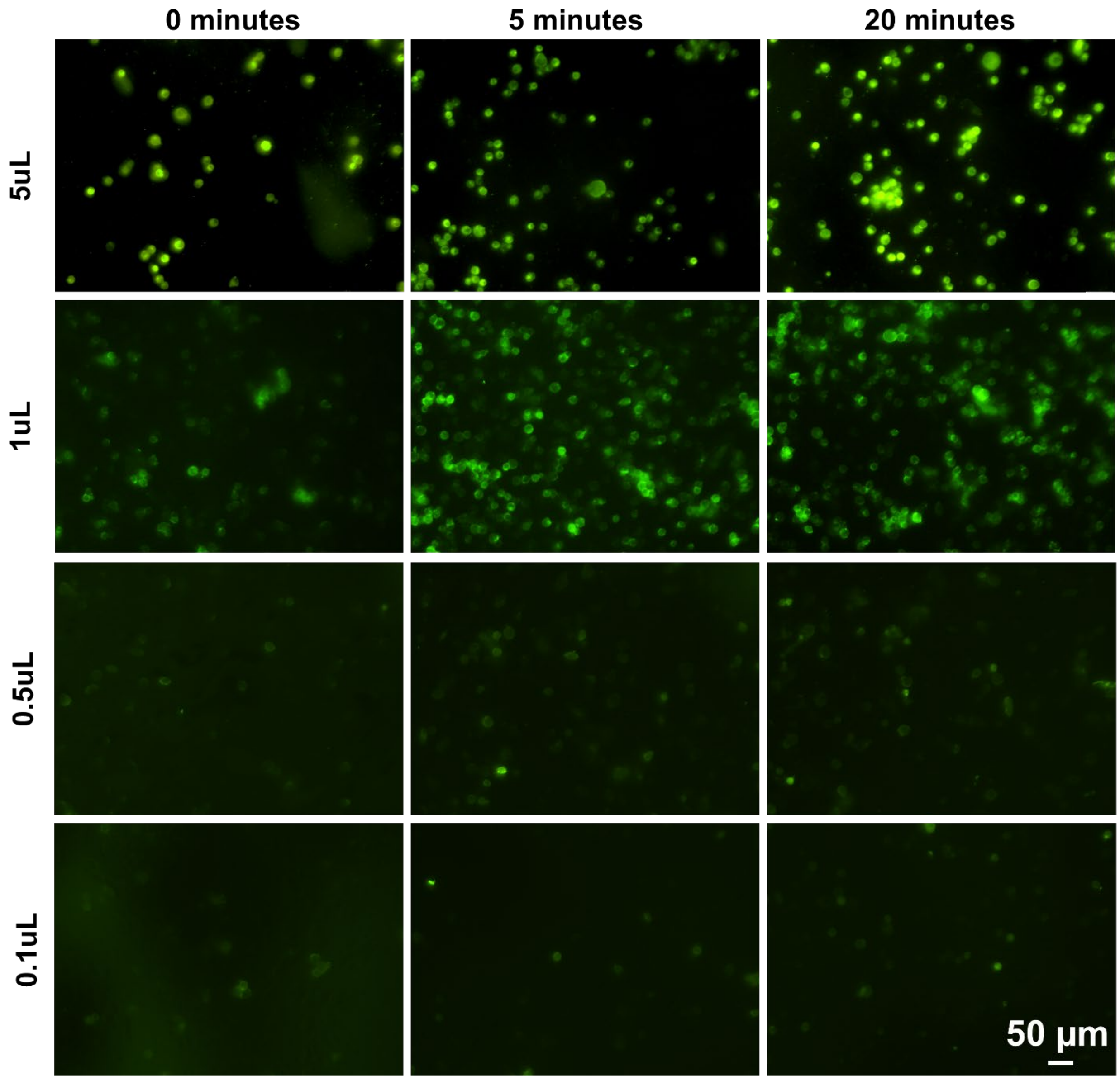

Figure S4. Characterisation of cytoplasmic dye concentration before using it for mixing experiment. The results showed that above 1 µL/million cells/mL, the cells are overstained from 0 minutes. Therefore 0.5 µL/million cells/mL was used to characterise the micromixer.

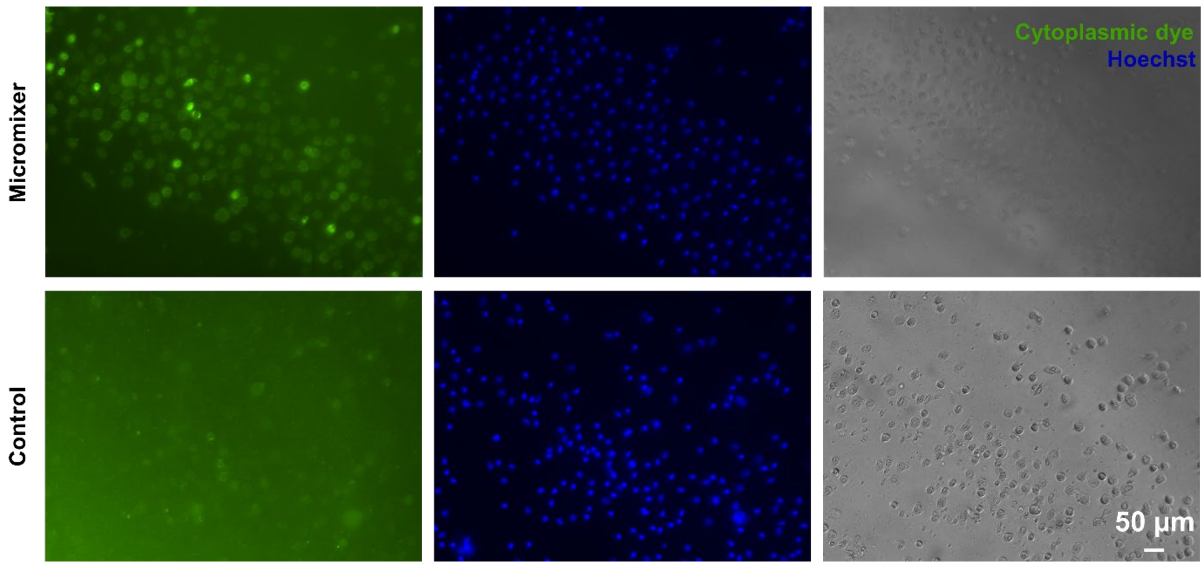

Figure S5 The micromixer mixing group and manual mixing group control cells after 15 minutes of staining. Results showed that most of the cells in micromixer groups are stained by both Hoechst and Cyotoplasmic dye, while many cells in the manual control groups are not stained with cytoplasmic dye.
